# Segmented regression modeling of a phytogenic blend on growth and immune function in broilers: a dose–response study

**DOI:** 10.1016/j.psj.2025.105913

**Published:** 2025-09-27

**Authors:** Hamid-Reza Behboodi, Morteza Asghari-Moghadam, Mehran Mehri

**Affiliations:** a Department of Animal and Poultry Physiology, Faculty of Animal Science, Gorgan University of Agricultural Science and Natural Resources; Gorgan 49138-15739, Iran; b Department of Animal Sciences, Faculty of Agriculture, University of Zabol; Sistan 98613-35856, Iran

**Keywords:** Dose–response, Hematology, Lymphoid organ, Medicinal herbs, Modeling

## Abstract

This study investigated the dose-dependent effects of a phytogenic feed additive, on growth performance, immune response, hematology, and organ development in broiler chickens. A total of 300-day-old broiler male chicks (Ross 308) with the average weight of 41.2 ± 2.32 g were randomly allocated into 4 treatment groups (0.0, 0.25, 0.50, and 1.00 mL/L phytogenic blend in drinking water) with 5 replications per groups (15 birds per pen) in a completely randomized design. Growth performance showed a clear bell-shaped response, with the 0.50 mL/L dose optimizing feed intake, body weight gain, and feed efficiency compared to the control and the highest dose. Humoral immunity was significantly enhanced at intermediate doses, where antibody titers against Newcastle and infectious bronchitis disease peaked. Similarly, key immunoglobulin concentrations were maximized at intermediate to high BMX levels. Hematological profiles also improved, with hemoglobin and leukocyte counts increasing at mid-to-high doses. Key stress indicators, such as the heterophil-to-lymphocyte ratio, were most favorable at intermediate doses. The development of crucial lymphoid organs (bursa and thymus) and concentrations of serum proteins also peaked within this intermediate-to-high dosage range. Multivariate analysis of all systemic variables confirmed that the 0.50 mL/L dose yielded the most beneficial immunological and physiological profile, while the 1.00 mL/L dose showed diminishing returns or adverse effects. In conclusion, BMX induced significant, dose-dependent benefits, with 0.50 mL/L emerging as the optimal inclusion level for enhancing performance, immunity, and systemic health in broilers.

## Introduction

The poultry industry continually seeks sustainable strategies to enhance broiler chicken performance, health, and welfare while reducing reliance on antibiotics. Phytogenic feed additives (PFAs), derived from medicinal herbs and plant extracts such as peppermint, thyme, licorice, sage, and eucalyptus, have emerged as promising natural alternatives due to their antimicrobial, antioxidant, and immunomodulatory properties (Aminullah et al., 2025; Ayalew et al., 2022). These plant-based compounds contain a diverse array of bioactive substances, such as essential oils, polyphenols, flavonoids, and terpenoids, which exhibit antimicrobial, antioxidant, anti-inflammatory, and immunomodulatory properties. Their inclusion in poultry diets has been associated with improvements in feed intake, nutrient digestibility, growth performance, and immune responses, contributing to healthier and more productive flocks (Windisch et al., 2008). Commonly studied individual herbs such as peppermint (*Mentha piperita*), thyme (*Thymus vulgaris*), licorice (*Glycyrrhiza glabra*), sage (*Salvia officinalis*), and eucalyptus (*Eucalyptus globulus*) have demonstrated beneficial effects in poultry through various mechanisms. For example, peppermint is known for their antimicrobial and digestive stimulant effects with benefits in improving meat quality, humoral immunity, and gut health in growing quails (Mehri et al., 2015a, 2015b). The essential oils from thyme and oregano, rich in thymol and carvacrol, are known for their potent antimicrobial and antioxidant activities that enhance gut health and nutrient absorption. Similarly, compounds like eucalyptol from *Eucalyptus* species are recognized for their mucolytic and bronchodilatory properties, supporting respiratory health. These individual components have been shown to improve growth performance, feed efficiency, and immune responses in broilers (Ayalew et al., 2022; Hesabi Nameghi et al., 2019; Wójcik et al., 2019), but there is limited information available on the effects of mixed herbal blends in poultry nutrition. Such combinations may offer synergistic benefits but also present challenges in determining optimal inclusion levels and understanding their comprehensive physiological impacts. The biological effects of PFAs often exhibit dose-dependent dynamics, with optimal supplementation maximizing benefits and excessive doses potentially diminishing efficacy or causing adverse effects (Aminullah et al., 2025; Mehri et al., 2015a, 2015b).

This study aimed to investigate the dose–dependent effects of a phytogenic feed additive formulated as a blend of peppermint, thyme, licorice, sage, and eucalyptus, on growth performance, immune parameters, hematological indices, and organ development in broiler chickens over a 42-day period. By applying segmented regression modeling and multivariate analyses, the research sought to identify optimal inclusion levels that maximize biological responses, providing evidence-based recommendations for the effective use of this blend in broiler nutrition.

## Materials and methods

### Ethics statement

This study protocol adheres to the guidelines established by the Iranian Council of Animal Care and it has been approved by the Research Animal Ethics Committee (AEUOZ-2012|UP-2020-BR) at the University of Zabol.

### Preparation of the BroncoMax

The BMX mixture, a blend of peppermint, thyme, licorice, sage, and eucalyptus, was carefully prepared to ensure a consistent final concentration of its components, and its physical and chemical properties were shown in Table 1. The product was administered on alternate days. This intermittent dosing strategy aligns with the manufacturer's protocol for practical, cost-effective application and is designed to maintain long-term efficacy by preventing physiological tolerance (Behboodi et al., 2021).

**Table 1:** Physical and chemical properties of BroncoMax solution.

Item	Amount
Specific Gravity (g/ml)	0.9901
Refractive Index	1.4390
pH	5.22
Density	1.004
Assay (Thymol)	0.984 %
Average Volume (ml)	1000 ml

### Birds, housing, and experimental design

A total of 300-day-old broiler male chicks (Ross 308) were obtained from a local hatchery with the average weight of 41.2 ± 2.32 g. The chicks were randomly allocated into 4 treatment groups with 5 replications per groups (15 birds per pen). A bell-shaped drinker and feeder were provided for each pen. The birds were raised on a concrete floor covered in wood shavings. Drinking water was the method of treatment administration; control (no BMX), and BMX concentrations of 0.25, 0.50, and 1.00 mL/L were added on alternating days, beginning on day 21. The birds had continuous access to food and water, receiving a commercial diet (Table 2) for the duration of the experiment. A 33°C temperature was used for a week, after which it was lowered by 2°C per week to 22°C. A continuous lighting protocol was used for 3 days, then changed to a 16L:8D cycle.

**Table 2:** Composition of basal diet.

Ingredient	Amount (%)		
	Starter	Grower	Finisher
Corn, Grain	52.50	49.06	52.50
Wheat, Red W.	20.61	15.00	20.61
Soybean Meal	18.98	26.75	18.98
Corn Gluten Meal	2.48	3.73	2.48
Limestone	1.44	1.42	1.44
DCP	1.31	1.47	1.31
Soybean Oil	1.00	1.09	1.00
NaHCO_3_	0.60	0.34	0.60
L-Lysine HCl	0.30	0.29	0.30
Vitamin Premix^1^	0.25	0.25	0.25
Mineral Premix^2^	0.25	0.25	0.25
DL-Methionine	0.15	0.20	0.15
NaCl	0.10	0.10	0.10
L-Thr	0.04	0.04	0.04
Nutrient specifications			
AME (kcal/kg)	2950	2950	2983
CP (%)	23.7	21.8	18.2
SID Lys (%)	1.20	1.10	0.91
SID M + C (%)	0.86	0.79	0.66
SID Thr (%)	0.76	0.69	0.57
SID Met (%)	0.56	0.51	0.41
Ca (%)	1.05	0.95	0.91
Available P (%)	0.50	0.45	0.40
DEB (mEq/kg)^3^	250	250	250

1 Provided the following per kilogram of diets: vitamin A (trans-retinyl acetate), 3600 IU; vitamin D3 (cholecalciferol), 800 IU; vitamin E (DL-a-tocopheryl acetate), 7.2 mg; vitamin K3, 1.6 mg; thiamine, 0.72 mg; riboflavin, 3.3 mg; niacin, 0.4 mg; pyridoxine, 1.2 mg; cobalamine, 0.6 mg; folic acid, 0.5 mg; choline chloride, 200 mg.

2 Provided the following per kilogram of diets: Mn (from MnSO4, H 2O), 40 mg; Zn (from ZnO), 40 mg; Fe (from FeSO4.7H2O), 20 mg; Cu (from CuSO4, 5H2O), 4 mg; I [from Ca (IO3)2H2O], 0.64 mg; Se (from sodium selenite), 0.08 mg.

3 Dieary electrolyte balance (Na^+^+ K^+^- Cl^-^).

### Performance parameters

To calculate body weight gain, feed intake, and feed conversion ratio, birds were weighed on a pen basis at hatch and 7, 14, 21, 35, and 42 days old. To get accurate feed conversion data, mortality was recorded when it happened. On day 42, four birds per pen (*n* = 20 birds per treatment) were selected, euthanized, and dissected to assess the relative weights of their internal organs. The liver, heart, and lymphoid organs (thymus, spleen, and bursa of Fabricius) were weighed and recorded.

### Vaccination program

All experimental chicks were subjected to a standardized vaccination protocol to ensure uniform protection against prevalent avian viral pathogens. On day 1 post-hatch, chicks were vaccinated via spray administration with a combination of infectious bronchitis virus (IB) vaccines, comprising both B1 and 793B strains. At 7 days of age, birds received a live Newcastle disease virus (ND) vaccine (LaSota strain) via ocular instillation. Concurrently, a bivalent inactivated vaccine targeting avian influenza (AI) and ND (Fatro, Italy) was administered intramuscularly. On day 14, a live intermediate strain vaccine against infectious bursal disease (IB) (GUMBO L, CEVA) was provided through the drinking water. To reinforce ND immunity, birds received an additional live attenuated ND vaccine (AVINEW, Merial) via drinking water on day 18. This vaccination regimen was designed to emulate commercial broiler production practices and to provide comprehensive immunological protection against major viral diseases.

### Blood collection and immunological assays

To evaluate humoral immune responses, blood samples from 4 birds per pen (*n* = 20 birds per treatment; 2 mL per bird) were aseptically collected from the brachial vein of each bird on days 21 and 35 post-hatch, corresponding to the primary and secondary immune responses, respectively. Samples were immediately centrifuged at 3,000 × *g* for 12 min to separate sera, which were then aliquoted and stored at −20°C until analysis. Serum antibody titers against ND were quantified using the hemagglutination inhibition (HI) assay, following the protocol described by Mehri et al. (2015b). Briefly, sera were serially diluted in V-bottom microtiter plates, and four hemagglutinating units of ND antigen were added to each well. After incubation, 1 % (v/v) chicken red blood cells were added, and the plates were examined for hemagglutination inhibition. Titers were expressed as the reciprocal of the highest serum dilution that completely inhibited hemagglutination. Antibody levels against IB virus were determined using a commercial enzyme-linked immunosorbent assay (ELISA) kit (Bionote, Korea; Kit no.: RG15-04), in accordance with the manufacturer’s instructions. Optical density was measured at 450 nm using a microplate reader, and antibody titers were calculated based on standard curves provided with the kit.

### Hematological and biochemical analyses

Following blood collection from 4 birds per pen (*n* = 20 birds per treatment), total red blood cells (RBCs) and white blood cells (WBCs) were enumerated using a hemocytometer. Hematocrit values were determined by the Wintrobe method, and hemoglobin concentrations were measured using the cyanmethemoglobin method, as described by Kececi et al. (1998). For differential leukocyte counts, blood smears were prepared from whole blood samples. The air-dried smears were stained with Wright-Giemsa stain (Saikin Kagaku Institute Co. Ltd, Sendai, Japan). Using a light microscope at 20 × magnification, white blood cells were counted until a total of 100 cells per slide was reached. The numbers of heterophil and lymphocyte were recorded, and the heterophil-to-lymphocyte (H:L) ratio was calculated. Plasma concentrations of total protein (TP) and albumin (ALB) were determined spectrophotometrically using a Stat Fax 3200 microplate reader.

### Statistical analysis

All data were analyzed using SAS version 9.4 (SAS Institute Inc., Cary, NC, USA). Growth performance, immunological, hematological, biochemical, and organ-weight parameters were subjected to one-way ANOVA with BMX inclusion level as the main effect. Where appropriate, polynomial contrasts were used to evaluate linear and quadratic dose–response trends. For variables measured at multiple time points, repeated-measures ANOVA was performed to assess main effects and treatment × time interactions. Pairwise comparisons among treatment means were conducted using Tukey’s post hoc test. Significance was declared at *P* < 0.05, and trends were discussed at 0.05 ≤ *P* < 0.10. To model dose–response relationships and estimate the optimal BMX inclusion for each biological endpoint, segmented (broken-line) regression analyses were performed, fitting linear–linear, quadratic–linear, and quadratic–quadratic two-slope models (Khosravi et al., 2016). Model fit was compared using goodness-of-fit statistics, as the most precise model had the highest R^2^ and lowest standard deviation of the residuals (*S_y.x_*):

$$S_{y.x}=\sqrt{\frac{SS}{df}}$$

Multivariate patterns among immunological, hematological, and organ-weight variables were explored using principal component analysis (PCA). The first two principal components were used to visualize clustering and to interpret the contribution of each variable to the observed variance. All results are presented as means ± standard error of the mean (SEM). Statistical analyses were visualized using GraphPad Prism (version 9.0) and R (version 4.2.0).

## Results

### Growth performance

BMX exerted a significant, dose-dependent effect on broiler performance (Table 3). Average feed intake increased (*P* < 0.001) up to 0.50 mL/L, reaching 124 g bird⁻¹ day⁻¹ (≈ 5 % higher than control), before declining at the highest inclusion level. Body-weight gain followed a similar quadratic pattern (*P* < 0.001), peaking at 70.9 g day⁻¹ with 0.50 mL/L—an increase of 7.3 % compared with the unsupplemented group. Feed conversion ratio (FCR) improved (*P* = 0.002) from 1.76 in controls to 1.72 at 0.50 mL/L, whereas birds given 1.00 mL/L showed the poorest FCR (1.81). No treatment × time interaction was detected for feed intake or gain, but FCR exhibited a modest interaction (*P* = 0.025)., and no pair-wise contrasts reached significance during the later growth phase or cumulatively.

**Table 3:** Effects of BroncoMax on performance of broiler chickens.

Response	BroncoMax (mL/L)				SEM	Probability		
	0.0	0.25	0.50	1.00		Treatment	Time	Treatment × Time
Feed intake (g)	118	122	124	121	0.392	<.0001	<.0001	0.879
Gain (g)	66.1	68.7	70.9	66.4	0.400	<.0001	<.0001	0.094
Feed conversion ratio	1.761	1.753	1.723	1.815	0.014	0.002	<.0001	0.025

Each value represents the average of 15 birds per pen (75 birds total per treatment).

### Humoral immunity, immunoglobulins and hematology

BMX supplementation augmented antibody production after ND vaccination and, to a lesser extent, IB vaccination (Table 4). ND haemagglutination-inhibition titers increased by 18 % at 21 d and 15 % at 35 d in birds, given 0.50 mL/L (linear and quadratic trends, *P* < 0.0001). IB responses were modest at 21 d (*P* = 0.054) but became significant by 35 d, again peaking at the mid-dose (*P* < 0.001). Serum IgT and IgM concentrations rose linearly at 21 d (*P* ≤ 0.001) and remained highest at 0.50–1.00 mL/L; by 35 d all three isotypes (Table 4) displayed significant dose effects, with IgT showing a pronounced quadratic response (*P* < 0.0001). Hematological indices (Table 5) were unchanged at 21 d except for modest increases in hematocrit and total leukocytes, but by 42 d hemoglobin (+35 %), hematocrit (+7 %), erythrocyte (+25 %) and leukocyte counts (+32 %) were elevated at the intermediate inclusion (linear *P* ≤ 0.004; quadratic for WBC, *P* = 0.001).

**Table 4:** Effects of BroncoMax on serum antibody titer for Newcastle disease virus (NDV) and infectious bronchitis disease (IBD) and serum concentrations of immunoglobulins (log_2_) at d 21 (Primary) and 35 (Secondary) of age.

Response	BroncoMax (mL/L)				SEM	Probability			
	0.0	0.25	0.50	1.00		Model	Linear	Quadratic	Control vs. BroncoMax
Primary NDV	4.25	4.90	5.01	4.72	0.063	<.0001	<.0001	<.0001	<.0001
Primary IBD	1776	1815	1844	1845	25.3	0.215	0.054	0.445	0.060
Secondary NDV	5.76	5.94	6.64	5.82	0.075	<.0001	0.018	<.0001	0.001
Secondary IBD	5929	6020	6293	6105	40.0	<.0001	0.001	0.003	0.001
Primary IgT	3.21	3.67	3.91	3.91	0.119	0.002	0.001	0.069	0.001
Primary IgY	1.28	1.29	1.18	1.37	0.089	0.524	0.694	0.318	0.989
Primary IgM	1.61	1.89	1.94	1.90	0.051	0.001	0.001	0.007	0.001
Secondary IgT	4.55	5.23	5.70	5.08	0.108	<.0001	0.001	<.0001	<.0001
Secondary IgY	1.76	1.98	2.28	2.09	0.089	0.007	0.006	0.040	0.004
Secondary IgM	2.16	2.45	2.81	2.60	0.059	<.0001	<.0001	0.001	<.0001

Each value represents the average of 4 birds per pen (20 birds total per treatment).

**Table 5:** Effects of BroncoMax on blood constitutes at d 21 and 42 of age.

Age	Response	BroncoMax (mL/L)				SEM	Probability			
		0.0	0.25	0.50	1.00		Model	Linear	Quadratic	Control vs. BroncoMax
21 d	Hb (g/dL)	7.61	7.40	7.95	7.67	0.210	0.358	0.453	0.884	0.810
	Hematocrit (%)	30.7	31.1	30.9	32.5	0.269	0.001	0.001	0.046	0.023
	RBC (10^6^/µl)	2.51	2.59	2.59	2.36	0.086	0.230	0.262	0.089	0.968
	WBC (10^3^/µl)	2.42	2.54	2.82	2.55	0.082	0.022	0.084	0.031	0.036
42 d	Hb (g/dL)	7.64	9.25	10.3	9.57	0.193	<.0001	<.0001	<.0001	<.0001
	Hematocrit (%)	31.4	32.7	33.5	32.8	0.341	0.004	0.004	0.008	0.001
	RBC (10^6^/µl)	2.67	2.93	3.35	3.02	0.072	<.0001	0.001	0.001	<.0001
	WBC (10^3^/µl)	2.69	3.19	3.56	3.04	0.108	0.001	0.009	0.001	0.001

Hb: hemoglobulin; RBC: red blood cells; WBC: white blood cells. Each value represents the average of 4 birds per pen (20 birds total per treatment).

### Stress indicators and serum proteins

BMX improved leukocyte differentials at 42 d, lowering heterophil percentage and the H:L ratio while raising lymphocyte percentage (*P* ≤ 0.001). Albumin and TP followed a strong quadratic pattern (*P* < 0.0001), each attaining maximal values, 52 % and 33 % above control, respectively, at 0.50 mL/L (Table 6).

**Table 6:** Effects of BroncoMax on heterophile (H; %), lymphocyte (L; %), H:L ratio, albumin (Alb) and total protein (TP) in serum at d 42 of age.

Response	BroncoMax (mL/L)				SEM	Probability			
	0.0	0.25	0.50	1.00		Model	Linear	Quadratic	Control vs. BroncoMax
H (%)	26.9	23.5	22.3	24.5	0.407	<.0001	0.001	<.0001	<.0001
L (%)	71.9	76.0	78.4	74.0	0.445	<.0001	0.001	<.0001	<.0001
H:L ratio	0.37	0.31	0.28	0.33	0.006	<.0001	<.0001	<.0001	<.0001
Alb (g/dL)	1.70	2.04	2.59	1.80	0.087	<.0001	0.042	<.0001	0.001
TP (g/dL)	3.74	4.43	4.99	3.91	0.092	<.0001	0.019	<.0001	<.0001

Hb: hemoglobulin; RBC: red blood cells; WBC: white blood cells. Each value represents the average of 4 birds per pen (20 birds total per treatment).

### Lymphoid and metabolic organs

Incremental inclusion of BMX produced organ-specific effects on relative weight (Table 7). The bursa responded most markedly, rising from 0.13 % in controls to a peak of 0.23 % at 0.50 mL/L before declining at the highest dose (quadratic *P* = 0.001; control vs treated *P* = 0.001). Thymus weight followed a similar curvilinear pattern, increasing to 0.26 % at 0.50 mL/L (model *P* = 0.002; quadratic *P* = 0.001), yet fell toward control values at 1.00 mL/L; overall treatment differed from control (*P* = 0.008). Spleen weight (0.14–0.16 %) was unaffected (*P* = 0.737). Liver weight tended to enlarge with supplementation, reaching 2.54 % at 0.50 mL/L (model *P* = 0.078; quadratic *P* = 0.050) and remained higher than control at all treated doses (control vs treated *P* = 0.014). Heart weight exhibited a modest quadratic response (model *P* = 0.042; quadratic *P* = 0.022), peaking at 0.55 % with 0.50 mL/L but not differing from control overall (*P* = 0.530). Collectively, BMX elicited significant, predominantly quadratic dose–response changes in lymphoid (bursa, thymus) and metabolic (liver, heart) organs, with maximal effects generally observed at 0.50 mL/L, while spleen mass remained stable.

**Table 7:** Effects of BroncoMax on the relative weight of internal organs, including bursa, thymus, spleen, liver, heart of the chickens at d 42 of age.

Response	BroncoMax (mL/L)				SEM	Probability			
	0.0	0.25	0.50	1.00		Model	Linear	Quadratic	Control vs. BroncoMax
Bursa (%)	0.13	0.19	0.23	0.17	0.012	0.001	0.013	0.001	0.001
Thymus (%)	0.20	0.25	0.26	0.21	0.010	0.002	0.477	0.001	0.008
Spleen (%)	0.14	0.16	0.15	0.15	0.011	0.737	0.875	0.299	0.421
Liver (%)	2.25	2.50	2.54	2.46	0.079	0.078	0.074	0.050	0.014
Heart (%)	0.52	0.53	0.55	0.51	0.009	0.042	0.689	0.022	0.530

Each value represents the average of 4 birds per pen (20 birds total per treatment).

### Dose–response modeling

**Fig. 1:**
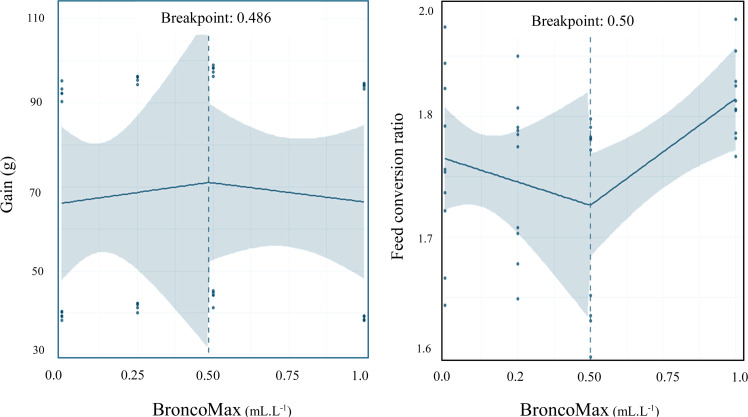
Dose-response relationship between BroncoMax levels (mL·L⁻¹) and gain (left) and feed conversion ratio (right) in broilers, modeled using two-slope segmented regressions: linear-ascending linear-descending.

*Performance*: Two-slope segmented regressions with two linear segments were fitted on gain and FCR data (Fig. 1). The estimated BMX level that maximized gain at 0.486 mL/L while minimized FCR at 0.50 mL/L, respectively.

**Fig 2:**
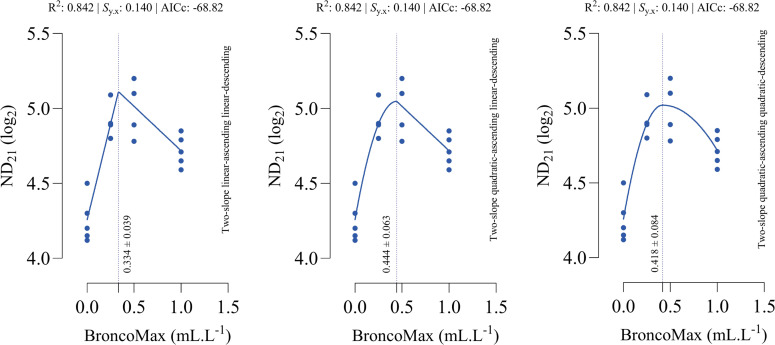
Dose-response relationship between BroncoMax levels (mL/L) and Newcastle disease titer at d 21 of age (ND_21_) in broilers, modeled using three two-slope segmented regressions: (left) linear-ascending linear-descending, (middle) quadratic-ascending linear-descending, and (right) quadratic-ascending quadratic-descending. Fitted models showed equal performance.

*ND_21_*: Across all three two-slope segmented regressions relating BMX inclusion to ND₂₁ titers in broilers, goodness-of-fit statistics were identical (Fig. 2), indicating equivalent explanatory power regardless of segment shape. The estimated BMX level that maximized ND₂₁ fell within a narrow, overlapping range of 0.334 mL/L for the linear–linear specification, 0.444 mL/L for the quadratic–linear specification, and 0.418 mL/L for the quadratic–quadratic specification.

**Fig 3:**
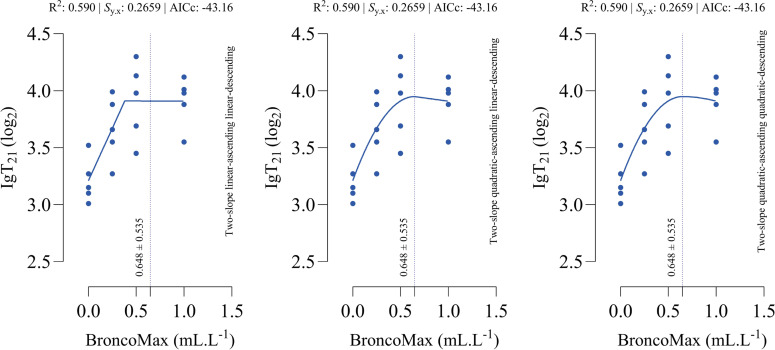
Dose-response relationship between BroncoMax levels (mL/L) and total immunoglobulins titer at d 21 of age (IgT_21_) in broilers, modeled using three two-slope segmented regressions: (left) linear-ascending linear-descending, (middle) quadratic-ascending linear-descending, and (right) quadratic-ascending quadratic-descending. Fitted models showed equal performance.

*IgT_21_*: All fitted models had the same goodness-of-fit (Fig. 3), and the optimal points (maximal) estimated by all three models was 0.648 mL/L at d 21 of age.

**Fig 4:**
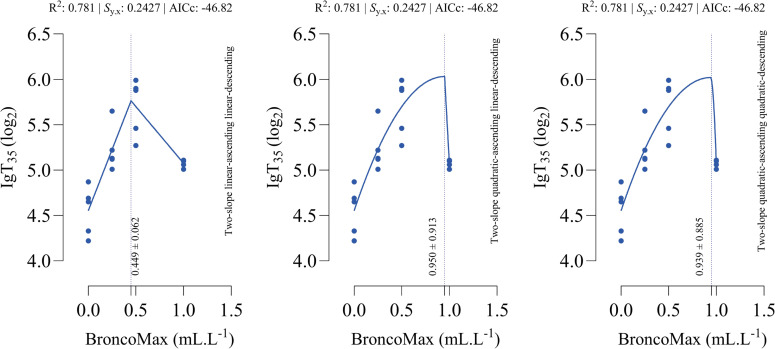
Dose-response relationship between BroncoMax levels (mL/L) and total immunoglobulins titer at d 35 of age (IgT_35_) in broilers, modeled using three two-slope segmented regressions: (left) linear-ascending linear-descending, (middle) quadratic-ascending linear-descending, and (right) quadratic-ascending quadratic-descending. Fitted models showed equal performance.

*IgT_35_*: All fitted models had the equal performance in terms of accuracy (Fig. 4). The optimal points for maximum IgT_35_, ranged from 0.449 mL/L for the linear–linear specification to 0.950 and 0.939 mL/L, for the quadratic–linear specification and the quadratic–quadratic specification, respectively.

**Fig 5:**
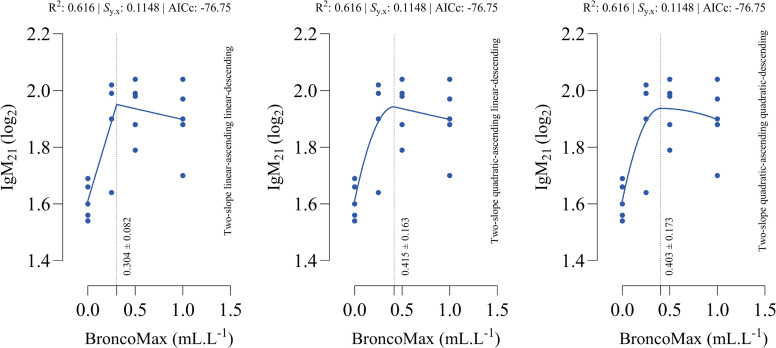
Dose-response relationship between BroncoMax levels (mL/L) and immunoglobulin M titer at d 21 of age (IgM_21_) in broilers, modeled using three two-slope segmented regressions: (left) linear-ascending linear-descending, (middle) quadratic-ascending linear-descending, and (right) quadratic-ascending quadratic-descending. Fitted models showed equal performance.

*IgM_21_*: All fitted models had the equal performance in terms of accuracy (Fig. 5). The optimal points for maximum IgM_21_ were estimated as 0.304, 0.415, and 0.403 mL/L for the linear–linear specification, the quadratic–linear specification, and the quadratic–quadratic specification, respectively.

**Fig 6:**
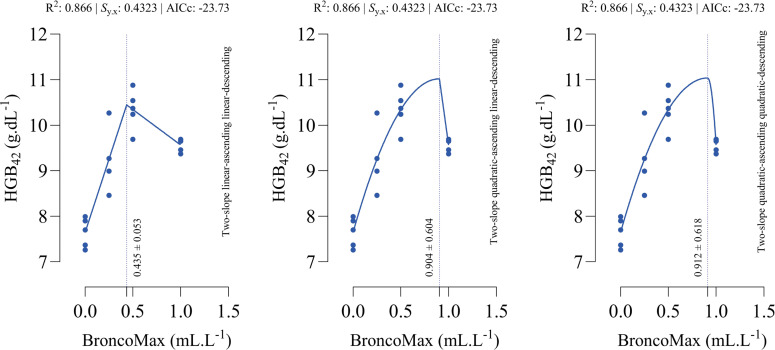
Dose-response relationship between BroncoMax levels (mL/L) and hemoglobin at d 42 of age (HGB_42_) in broilers, modeled using three two-slope segmented regressions: (left) linear-ascending linear-descending, (middle) quadratic-ascending linear-descending, and (right) quadratic-ascending quadratic-descending. Fitted models showed equal performance.

*HGB_42_*: All fitted models had the equal performance in terms of accuracy (Fig. 6). The optimal points for maximum HGB_42_ were estimated as 0.435, 0.904, and 0.912 mL/L for the linear–linear specification, the quadratic–linear specification, and the quadratic–quadratic specification, respectively.

**Fig 7:**
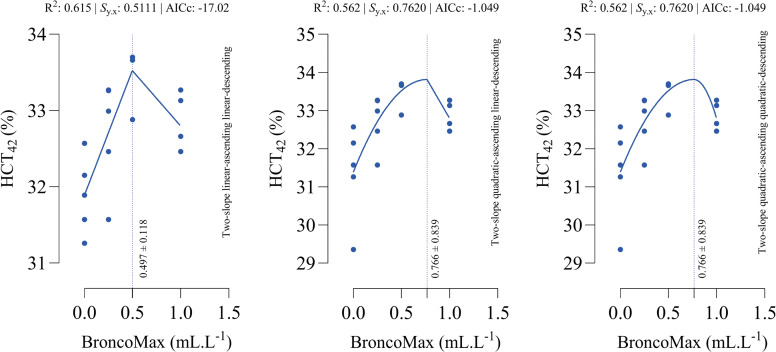
Dose-response relationship between BroncoMax levels (mL/L) and hematocrit at d 42 of age (HCT_42_) in broilers, modeled using three two-slope segmented regressions: (left) linear-ascending linear-descending, (middle) quadratic-ascending linear-descending, and (right) quadratic-ascending quadratic-descending. The linear-ascending linear-descending model outperformed other models.

*HTC_42_*: Although two-slope quadratic-ascending linear-descending and quadratic-ascending quadratic-descending models had the same goodness-of-fit statistics, the linear-ascending linear-descending model outperformed the other models (Fig. 7). The optimal points for maximum HTC_42_ were estimated as 0.497, 0.766, and 0.766 mL/L for the linear–linear specification, the quadratic–linear specification, and the quadratic–quadratic specification, respectively.

**Fig 8:**
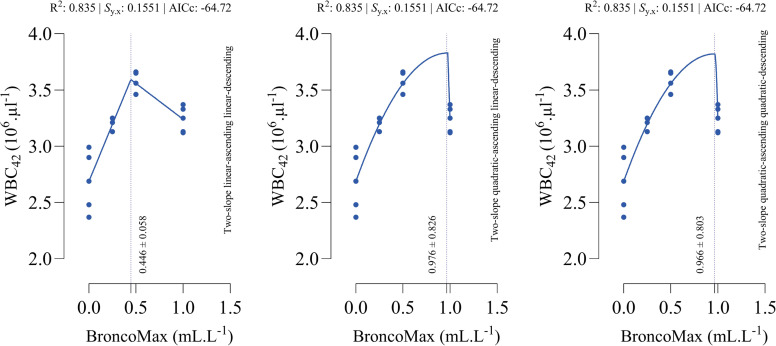
Dose-response relationship between BroncoMax levels (mL/L) and white blood cells at d 42 of age (WBC_42_) in broilers, modeled using three two-slope segmented regressions: (left) linear-ascending linear-descending, (middle) quadratic-ascending linear-descending, and (right) quadratic-ascending quadratic-descending. Fitted models showed equal performance.

*WBC_42_*: All fitted models had the equal performance in terms of accuracy (Fig. 8). The optimal points for maximum WBC_42_ were estimated as 0.446, 0.976, and 0.966 mL/L for the linear–linear specification, the quadratic–linear specification, and the quadratic–quadratic specification, respectively.

**Fig 9:**
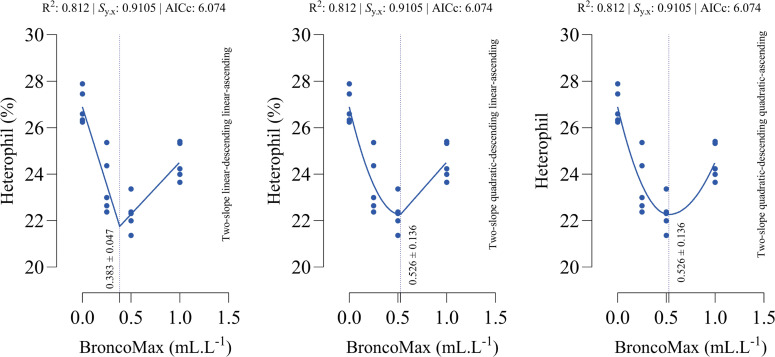
Dose-response relationship between BroncoMax levels (mL/L) and heterophil in broilers, modeled using three two-slope segmented regressions: (left) linear-ascending linear-descending, (middle) quadratic-ascending linear-descending, and (right) quadratic-ascending quadratic-descending. Fitted models showed equal performance.

*Heterophil count*: All fitted models had the equal performance in terms of accuracy (Fig. 9). The optimal points for minimum heterophil count were estimated as 0.383, 0.526, and 0.526 mL/L for the linear–linear specification, the quadratic–linear specification, and the quadratic–quadratic specification, respectively.

**Fig 10:**
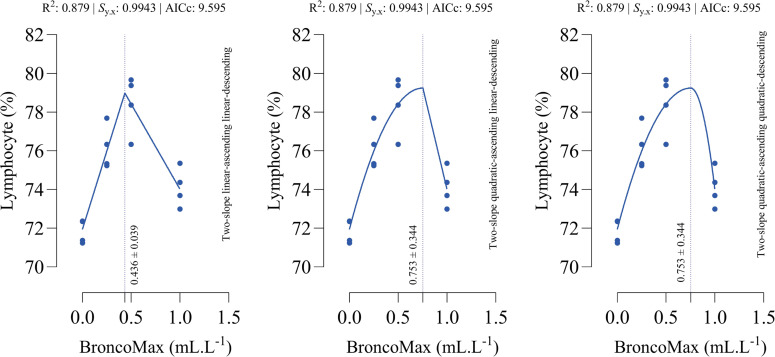
Dose-response relationship between BroncoMax levels (mL/L) and lymphocyte in broilers, modeled using three two-slope segmented regressions: (left) linear-ascending linear-descending, (middle) quadratic-ascending linear-descending, and (right) quadratic-ascending quadratic-descending. Fitted models showed equal performance.

*Lymphocyte count*: All fitted models had the equal performance in terms of accuracy (Fig. 10). The optimal points for maximum lymphocyte count were estimated as 0.436, 0.753, and 0.753 mL/L for the linear–linear specification, the quadratic–linear specification, and the quadratic–quadratic specification, respectively.

**Fig. 11:**
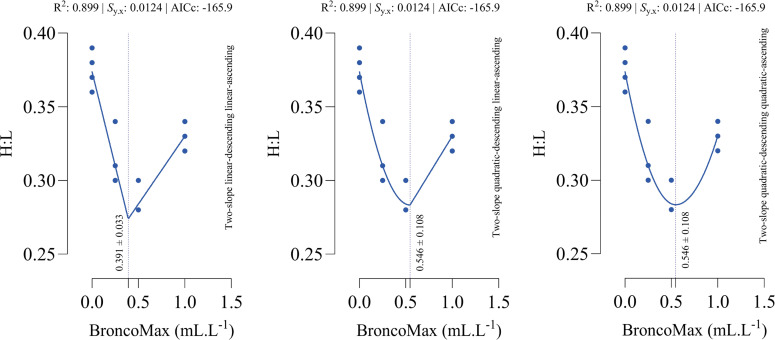
Dose-response relationship between BroncoMax levels (mL/L) and heterophil to lymphocyte ratio (H:L) in broilers, modeled using three two-slope segmented regressions: (left) linear-descending linear-ascending, (middle) quadratic-descending linear-ascending, and (right) quadratic-descending quadratic-ascending. Fitted models showed equal performance.

*H:L ratio*: All fitted models had the equal performance in terms of accuracy (Fig. 11). The optimal points for minimum H:L ratio were estimated as 0.391, 0.546, and 0.546 mL/L for the linear–linear specification, the quadratic–linear specification, and the quadratic–quadratic specification, respectively.

**Fig. 12:**
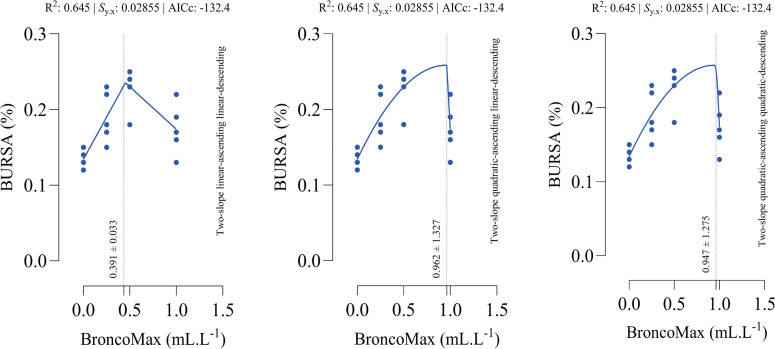
Dose-response relationship between BroncoMax levels (mL/L) and bursa weight (% of body weight) in broilers, modeled using three two-slope segmented regressions: (left) linear-ascending linear-descending, (middle) quadratic-ascending linear-descending, and (right) quadratic-ascending quadratic-descending. Fitted models showed equal performance.

*Bursa*: All fitted models had the equal performance in terms of accuracy (Fig. 12). The optimal points for maximum relative weight of bursa were estimated as 0.391, 0.962, and 0.947 mL/L for the linear–linear specification, the quadratic–linear specification, and the quadratic–quadratic specification, respectively.

**Fig. 13:**
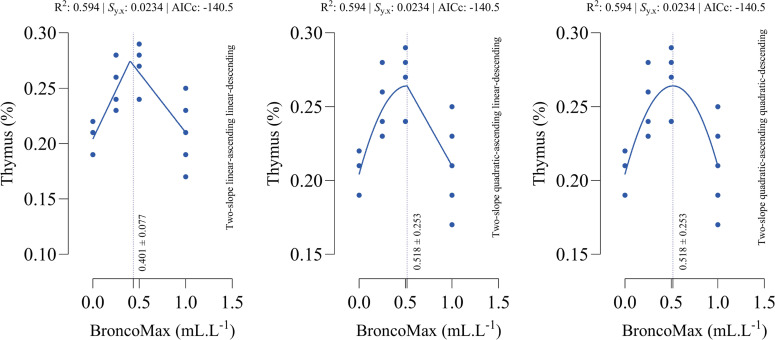
Dose-response relationship between BroncoMax levels (mL/L) and thymus weight (% of body weight) in broilers, modeled using three two-slope segmented regressions: (left) linear-ascending linear-descending, (middle) quadratic-ascending linear-descending, and (right) quadratic-ascending quadratic-descending. Fitted models showed equal performance.

*Thymus*: All fitted models had the equal performance in terms of accuracy (Fig. 13). The optimal points for maximum relative weight of thymus were estimated at 0.401, 0.518, and 0.518 mL/L for the linear–linear specification, the quadratic–linear specification, and the quadratic–quadratic specification, respectively.

**Fig. 14:**
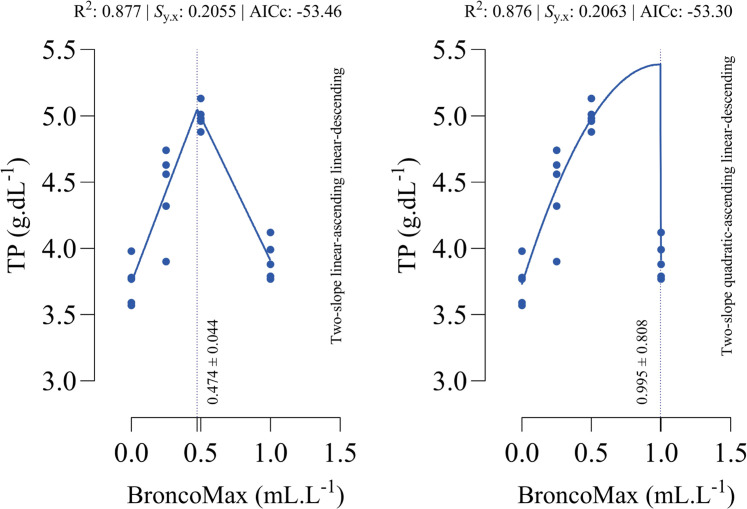
Dose-response relationship between BroncoMax levels (mL/L) and serum total protein (TP, g.dL^-1^) in broilers, modeled using three two-slope segmented regressions: (left) linear-ascending linear-descending, (right) quadratic-ascending linear-descending. The linear-ascending linear-descending model had better performance than the other model.

*TP*: Two models of two-slope linear-ascending linear-descending and quadratic-ascending linear-descending were fitted, and former model was slightly better than the other one (Fig. 14). The optimal points for maximum relative weight of bursa were estimated at 0.474 and 0.995 mL/L for the linear–linear specification and the quadratic–linear specification, respectively.

### Multivariate confirmation

**Fig. 15:**
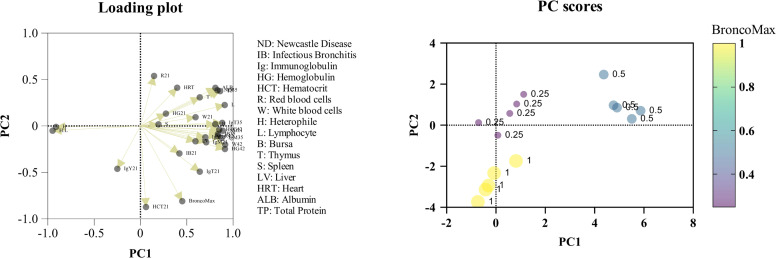
Principal-component analysis of systemic immune and physiological responses to graded dietary BroncoMax in broiler chickens. Loading plot: vectors represent variable loadings on PC1 and PC2 (eigenvalues = 14.67 and 3.27, respectively), with arrow length proportional to contribution; clustered arrows for antibody titers (ND, IB, Ig) and leukocyte counts define the positive PC1 axis, whereas opposite vectors (e.g., H:L, BroncoMax concentration) indicate negative correlations. Score plot: each dot is pen replicate, colored and sized by BroncoMax level (0.25 %, 0.5 %, 1 %); dashed lines mark the multivariate origin. The 0.5 % group occupies the extreme positive PC1 quadrant, the 0.25 % group lies near the origin with slightly elevated PC2, and the 1 % group shifts to negative PC1/PC2, illustrating a bell-shaped dose response.

As shown in Table 8, the first principal component (PC1) explained the largest proportion of variance and was characterized by strong positive loadings for ND_21_, ND_35_, IB_35_, IgT_35_, HG_42_, HTC_42_, R_42_ (RBC_42_), W_42_ (WBC_42_), L (lymphocyte), B (bursa), Alb, and TP, indicating that these variables contribute most substantially to the primary axis of variation. Conversely, H and H:L ratio displayed strong negative loadings on PC1, suggesting an inverse relationship with overall performance and health markers. The second principal component (PC2) differentiated variables such as BroncoMax and HCT_21_, both of which exhibited strong negative loadings, while ND_35_ and R_21_ (RBC_21_) showed moderately positive associations. These patterns highlight that both immune function and stress indices are major drivers of the observed variation, with BroncoMax distinctly influencing PC2 (Fig. 15).

**Table 8:** Principal component analysis (PCA) loadings for the immunological and hematological variables.

Variable	PC1	PC2
BroncoMax	0.450915802720051	−0.809934817336404
ND_21_	0.888648793484008	−0.0516242536595936
ND_35_	0.831410190369905	0.383691737659243
IB_21_	0.420088018680465	−0.296053868925077
IB_35_	0.836093938555409	−0.0875209367586875
IgT_21_	0.640088306874558	−0.492407795262381
IgY_21_	−0.249819397346316	−0.461484452733999
IgM_21_	0.709140570744008	−0.175698031149897
IgT_35_	0.882760420529994	0.0328667872206415
IgY_35_	0.699828563412843	−0.122574025738529
IgM_35_	0.877178424202356	−0.127956008494462
HG_21_	0.274668918572404	0.132805124279128
HCT_21_	0.0593387158306817	−0.87291612318063
R_21_	0.146430852686011	0.538547469557069
W_21_	0.5945023118	0.0941481635934483
HG_42_	0.91159083187723	−0.248205766711177
HTC_42_	0.850222465349281	−0.0400568169316674
R_42_	0.834994477929718	−0.109811205463053
W_42_	0.913947255861009	−0.200379609613424
H	−0.911423258698682	−0.0102810959535008
L	0.909167210008558	0.22524971098498
H:L	−0.948778376624073	−0.0513349959682317
B	0.80721193862076	0.019627010774818
T	0.639675299339652	0.308303751466815
S	0.194299213442668	0.0167287484128701
LV	0.593198502143231	−0.164817040467295
HRT	0.395904418319491	0.411303405329602
ALB	0.808575109740446	0.410427096240182
TP	0.860162131908159	0.375115975154814

ND_21_: Newcastle disease antibody measured at d 21 posthatch; ND_35_: Newcastle disease antibody measured at d 35 posthatch; IB_21_: infectious bronchitis antibody measured at d 21 posthatch; IB_35_: infectious bronchitis antibody measured at d 35 posthatch; IgT_21_: total immunoglobulin measured at d 21 posthatch; IgY_21_: immunoglobulin Y measured at d 21 posthatch; IgM_21_: immunoglobulin M measured at d 21 posthatch; IgT_35_: total immunoglobulin measured at d 35 posthatch; IgY_35_: immunoglobulin Y measured at d 35 posthatch; IgM_35_: immunoglobulin M measured at d 35 posthatch; HG_21_: hemoglobin measured at d 21 posthatch; HCT_21_: hematocrit measured at d 21 posthatch; R_21_: red blood cells measured at d 21 posthatch; W_21_: white blood cells measured at d 21 posthatch; HG_42_: hemoglobin measured at d 42 posthatch; HTC_42_: hematocrit measured at d 42 posthatch; R_42_: red blood cells measured at d 42 posthatch; W_42_: white blood cells measured at d 42 posthatch; H: heterophil; L: lymphocyte; H:L: heterophil: lymphocyte ratio; B: bursa; T: thymus; S: spleen; LV: liver; HRT: heart; ALB: albumin; TP: total protein.

## Discussion

The modern poultry industry continually seeks strategies to optimize broiler health, productivity, and welfare, while reducing reliance on antibiotics. Phytogenic feed additives (PFAs), derived from plant sources, have emerged as promising alternatives due to their multifaceted effects on growth, immunity, and stress resilience (Fascina et al., 2017; Onunkwo et al., 2020). BMX, a novel phytogenic additive, was evaluated in a dose-response study to elucidate its impact on broiler performance, immunity, hematology, organ development, and physiological stress.

BMX supplementation exerted a dose-dependent effect on broiler growth performance. Feed intake increased significantly up to 0.50 mL/L, peaking at 124 g/b/d—about 5 % higher than the control group. This trend followed a quadratic pattern, with intake declining at the highest tested dose. Body-weight gain mirrored this trajectory, reaching a maximum of 70.9 g/d at the 0.50 mL/L inclusion level, representing a 7.3 % improvement over unsupplemented birds. These findings align with previous studies reporting that moderate supplementation with phytogenic additives can enhance both feed intake and weight gain, likely due to improved palatability, gut health, and nutrient absorption (Onunkwo et al., 2020). However, excessive inclusion may lead to reduced performance, possibly due to metabolic overload, palatability issues, or negative feedback mechanisms (Fascina et al., 2017). Feed conversion ratio improved from 1.761 in controls to 1.723 at 0.50 mL/L, indicating greater efficiency in converting feed into body mass. Notably, birds receiving the highest dose (1.00 mL/L) exhibited the poorest FCR (1.815), underscoring the importance of optimal dosing. The quadratic relationship between BMX inclusion and FCR reflects the bell-shaped response commonly observed with nutritional interventions (Wang et al., 2019). The improvement in FCR is particularly significant from an economic and environmental perspective, as feed costs constitute the largest proportion of production expenses, and efficient feed utilization reduces waste and resource use (Hippenstiel et al., 2011). Overall, PFAs enhance the apparent ileal digestibility of nutrients, such as crude protein and amino acids, which leads to better nutrient absorption in the small intestine. For instance, studies have shown that broilers fed diets supplemented by PFAs exhibited improved digestibility of crude protein and ether extract compared to control groups (Amad et al., 2011). The inclusion of PFAs has been linked to increased secretion of digestive enzymes, which further aids in nutrient breakdown and absorption (Hafeez et al., 2016), resulting in better performance of broilers fed PFAs like BMX.

BMX supplementation augmented humoral immunity, as evidenced by increased antibody production following ND and IB vaccinations. ND haemagglutination-inhibition titers rose by 18 % at 21 days and 15 % at 35 days in birds given 0.50 mL/L, with both linear and quadratic trends highly significant. IB responses were modest at 21 days but became significant by 35 days, again peaking at the mid-dose. These enhancements suggest that BMX may act as an immunomodulator, potentiating vaccine-induced antibody production. This is consistent with literature reporting that phytogenic feed additives, such as essential oils of thyme and star anise (Amad et al., 2011), chromium methionine supplementation under stress conditions (Ebrahimzadeh et al., 2012), and plant extracts including turmeric, citrus, grape seed, cinnamon, boldo, and fenugreek (Fascina et al., 2017), can stimulate both humoral and cellular immune responses in poultry. Serum concentrations of IgT and IgM increased linearly at 21 days and remained highest at 0.50–1.00 mL/L. By 35 days, all three major isotypes showed significant dose effects, with IgT exhibiting a pronounced quadratic response. Enhanced immunoglobulin synthesis is indicative of a more robust adaptive immune system, which is critical for disease resistance and vaccine efficacy. One of the key mechanisms through which PFAs exert their beneficial effects is the modulation of the immune system within the gastrointestinal tract. Studies have demonstrated that PFAs can stimulate both innate and adaptive immune responses in the gut, leading to enhanced intestinal health and improved overall performance in broiler chickens (Windisch et al., 2008; Yang et al., 2015). For instance, supplementation with PFAs has been associated with significant morphological improvements in the small intestine. Wang et al. (2021) reported that broilers fed with diets enriched with PFAs exhibited increased villus height and villus height-to-crypt depth ratios in the jejunum and ileum. These morphological changes are indicative of improved absorptive surface area, which enhances the bird's ability to digest and absorb nutrients more efficiently. Enhanced nutrient absorption not only supports better feed conversion but also contributes to overall growth and health status. Moreover, PFAs have been shown to modulate the expression of immune-related genes in the intestinal mucosa, particularly in the ileum. Pirgozliev et al. (2019) observed that PFA supplementation led to the upregulation of key cytokines and pattern recognition receptors involved in immune surveillance and inflammation control.

Hematological indices were largely unchanged except of modest increases in hematocrit and total leukocyte counts in early period of growth. However, at the later period of growth, birds at the intermediate BMX inclusion level exhibited marked increases in hemoglobin (+35 %), hematocrit (+7 %), erythrocyte (+25 %), and leukocyte counts (+32 %). These changes suggest improved oxygen-carrying capacity and immune cell availability, both of which are helpful for growth and disease resistance. Similar trends have been reported in broilers supplemented with various PFAs and probiotics, where moderate inclusion levels enhance hematological health, but excessive doses may yield diminishing or adverse effects (Onunkwo et al., 2020). A lower H:L ratio is widely recognized as a marker of reduced physiological stress in poultry. The observed increases in lymphocyte counts further support the immunostimulatory properties of BMX.

Albumin and total protein (TP) levels followed a strong quadratic pattern, peaking at 0.50 mL/L with values 52 % and 33 % above control, respectively. Elevated serum protein levels reflect improved nutritional status and protein metabolism, and may also be linked to enhanced immune function. Furthermore, increasing dietary levels of BMX supplementation led to organ-specific changes in lymphoid tissues; for example, the relative weight of the bursa of Fabricius increased from 0.13 % in the control group to 0.23 % at 0.50 mL·L⁻¹ of BMX, but decreased again at the highest inclusion level. Thymus showed a similar curvilinear pattern, peaking at 0.26 % at 0.50 mL/L, while spleen remained unaffected (0.14–0.16 %). The bursa of Fabricius and the thymus are essential organs in the development of B and T lymphocytes, respectively, playing critical roles in the avian immune system (Weissman et al., 1993). Hematopoietic stem cells migrate to the bursa, where they undergo a process of selection and maturation. The bursal environment influences the expression of specific surface markers that characterize B cells (Boyd et al., 1983). The bursa also produces glucocorticoids, which are important for B cell selection and maturation (Lechner et al., 2001). The thymus is crucial for the development of T lymphocytes. It serves as the site where T cell precursors mature and learn to recognize self-antigens, a process essential for establishing immune tolerance (Miller, 2020). T cell precursors migrate from the bone marrow to the thymus, where they undergo differentiation and selection processes. This includes the expression of T cell-specific surface markers and the ability to recognize antigens presented by major histocompatibility complex (MHC) molecules (Goher et al., 2024). The thymus also plays a role in the education of T cells to ensure that they do not react against the body's own tissues (Cooper et al., 1966). Phytogenic substances, including flavonoids and phenolic compounds, possess antioxidative properties that can mitigate oxidative stress and inflammation in animals. Chronic inflammation and oxidative stress can lead to cellular damage and apoptosis, affecting the immune system's functionality, including the bursa and thymus (Basiouni et al., 2023). These herbal compounds can enhance the immune response by modulating the activities of immune cells. For instance, certain phytogenic products have been reported to increase the production of antibodies and improve the overall immune status of animals, which may involve the bursa and thymus as key sites for immune cell development and maturation (Hashemipour et al., 2013). The enlargement of bursa and thymus at optimal BMX doses suggests enhanced lymphopoiesis and immune competence. These findings are consistent with reports that PFAs can stimulate lymphoid organ growth, thereby supporting systemic immunity (Fascina et al., 2017). On the other hand, the metabolic organs such as liver and heart tended to enlarge with supplementation, reaching 2.54 and 0.55 % at 0.50 mL/L, respectively. The liver is pivotal for metabolism and detoxification, and its enlargement may reflect increased metabolic activity or enhanced nutrient processing (Grant, 1991), or accompanied by changes in the expression of genes involved in metabolic pathways, indicating a shift in liver function to accommodate increased processing needs (Navarro et al., 2009). However, less pronounced changes in relative heart weight suggest minimal cardiovascular effects within the tested dose range.

To identify the BMX concentration that optimizes each biological endpoint, segmented (broken-line) regression models were employed. This approach is particularly suitable for responses that rise rapidly to a threshold and then decline—a pattern observed for many physiological and performance traits in nutrition studies (Ahmadi et al., 2025; Ghazaghi et al., 2024b). The dose–response modeling reveals that BMX exerts dose-dependent benefits across a wide array of physiological systems in broiler chickens. Optimal ranges for immunological parameters tend to cluster between 0.3–0.9 mL/L, with specific indices like TP and WBC maintaining responsiveness across a broader spectrum. Conversely, stress mitigation and lymphoid organ development require more tightly defined dosages. These findings suggest that BMX can be strategically dosed to support immune health, stress resilience, and hematological performance in commercial broiler production. Further research may refine precise timing and dose adjustments depending on age, production phase, or disease challenge status to maximize benefits while maintaining cost-effectiveness and animal welfare.

This study demonstrates that BMX supplementation modulates immune, hematological, and organ-weight parameters in broilers in a dose-dependent manner, as revealed by PCA. The bell-shaped dose-response, with 0.5 % BMX optimally enhancing both humoral and cellular immunity, is consistent with findings from recent peer-reviewed studies on phytogenic feed additives and dietary supplements in poultry. Moderate supplementation of phytogenic or functional additives has been shown to improve immune responses and growth performance, while excessive doses may lead to immunosuppression or reduced productivity (Ramah et al., 2020; Ren et al., 2025; Wang et al., 2021). The separation of treatment groups along PC1, driven by antibody titers, leukocyte counts, and hemoglobin levels, mirrors results from studies where optimal additive dosing increased antibody production and supported lymphoid organ development (Abdelli et al., 2021; Ren et al., 2025). The negative association of stress markers (such as the H:L ratio) with PC1 further confirms the inverse relationship between stress and performance, as established in the literature (Kim et al., 2021; Oni et al., 2024). Notably, the distinct clustering of BroncoMax on PC2 suggests targeted modulation of specific physiological pathways, such as antioxidant defense or organ integrity, a phenomenon also observed with certain phytogenic and functional feed additives (Al-Garadi et, al.; Ren et al., 2025). The application of PCA in this context provides a robust multivariate approach to integrate immune, hematological, and performance data, allowing for the identification of key factors influencing health and productivity in animal nutrition research (Asghari-Moghadam et al., 2024; Ghazaghi et al., 2024a; Zhao et al., 2021). Collectively, these findings reinforce the importance of precise dosing for dietary supplements like BMX to optimize immune function and stress resilience in poultry, while highlighting the potential risks of excessive supplementation.

In conclusion, this study demonstrates that BMX, a phytogenic feed additive composed of peppermint, thyme, licorice, sage, and eucalyptus oil, exerts dose-dependent benefits on broiler performance, immunity, stress resilience, and organ development. A range of optimal inclusion levels of BMX (approximately 0.3–0.6 mL/L) enhanced feed intake, growth rate, and feed efficiency, antibody responses to ND and IB vaccines, serum immunoglobulin concentrations, and lymphoid organ development. Hematological indices such as hemoglobin and leukocyte counts, improved at moderate doses, accompanied by reduced H:L ratios and elevated total protein levels, indicating better physiological and immune status. Principal component analysis confirmed the bell-shaped response pattern, with 0.5 mL/L emerging as the most effective dose for enhancing both humoral and cellular immune functions without eliciting adverse metabolic effects. Segmented regression further supported this threshold-based efficacy, highlighting the need for precise dose optimization.

## CRediT authorship contribution statement

**Hamid-Reza Behboodi:** Writing – review & editing, Investigation. **Morteza Asghari-Moghadam:** Writing – review & editing, Funding acquisition, Conceptualization. **Mehran Mehri:** Formal analysis, Writing – original draft, Writing – review & editing.

## Disclosures

The authors declare that they have no known competing financial interests or personal relationships that could have appeared to influence the work reported in this paper.
